# Dysfunction of the energy sensor NFE2L1 triggers uncontrollable AMPK signaling and glucose metabolism reprogramming

**DOI:** 10.1038/s41419-022-04917-3

**Published:** 2022-05-25

**Authors:** Lu Qiu, Qiufang Yang, Wenshan Zhao, Yadi Xing, Peng Li, Xiaowen Zhou, Haoming Ning, Ranran Shi, Shanshan Gou, Yalan Chen, Wenjie Zhai, Yahong Wu, Guodong Li, Zhenzhen Chen, Yonggang Ren, Yanfeng Gao, Yiguo Zhang, Yuanming Qi

**Affiliations:** 1 School of Pharmaceutical Sciences (Shenzhen), Sun Yat-sen University, Shenzhen, 518107 China; 2 School of Life Sciences, Zhengzhou University, Zhengzhou, 450001 China; 3 The Laboratory of Cell Biochemistry and Topogenetic Regulation, College of Bioengineering and Faculty of Sciences, Chongqing University, Chongqing, 400044 China; 4 School of Agricultural Sciences, Zhengzhou University, Zhengzhou, 450001 China; 5 Department of Biochemistry, North Sichuan Medical College, Nanchong, 637000 China; 6 Henan Key Laboratory of Bioactive Macromolecules, Zhengzhou University, Zhengzhou, 450001 China; 7 International Joint Laboratory for Protein and Peptide Drugs of Henan Province, Zhengzhou University, Zhengzhou, 450001 China

**Keywords:** Nutrient signalling, Transcription factors, Metabolic pathways

## Abstract

The antioxidant transcription factor NFE2L1 (also called Nrf1) acts as a core regulator of redox signaling and metabolism homeostasis, and thus, its dysfunction results in multiple systemic metabolic diseases. However, the molecular mechanism(s) by which NFE2L1 regulates glycose and lipid metabolism remains elusive. Here, we found that loss of NFE2L1 in human HepG2 cells led to a lethal phenotype upon glucose deprivation and NFE2L1 deficiency could affect the uptake of glucose. Further experiments revealed that glycosylation of NFE2L1 enabled it to sense the energy state. These results indicated that NFE2L1 can serve as a dual sensor and regulator of glucose homeostasis. The transcriptome, metabolome, and seahorse data further revealed that disruption of NFE2L1 could reprogram glucose metabolism to aggravate the Warburg effect in NFE2L1-silenced hepatoma cells, concomitant with mitochondrial damage. Co-expression and Co-immunoprecipitation experiments demonstrated that NFE2L1 could directly interact and inhibit AMPK. Collectively, NFE2L1 functioned as an energy sensor and negatively regulated AMPK signaling through directly interacting with AMPK. The novel NFE2L1/AMPK signaling pathway delineate the mechanism underlying of NFE2L1-related metabolic diseases and highlight the crosstalk between redox homeostasis and metabolism homeostasis.

## Introduction

Redox homeostasis is a necessary prerequisite for the maintenance of physiological responses, and the imbalance in redox homeostasis leads to various chronic systemic diseases. Nuclear factor erythroid 2 like 1 (NFE2L1/Nrf1), a member of the cap’n’collar basic-region leucine zipper (CNC-bZIP) family, plays a critical role in regulating redox homeostasis [1–4]. Studies have revealed that NFE2L1 plays an important role in metabolic pathways and influences the development of metabolic diseases. The single nucleotide polymorphism on the 5′-flanking regions of the *NFE2L1* has been associated with obesity [5]. The overexpression of *NFE2L1* eventually developed into diabetes mellitus in transgenic mice [6]. In mouse pancreatic β cells, knockout of *NFE2L1* causes hyperinsulinemia and glucose intolerance [7]. In adipocytes, knockout of *NFE2L1* results in disappearance of subcutaneous adipose tissue, accompanied by insulin resistance, adipocyte hypertrophy, and obesity-related inflammation [8]. Knockout of *NFE2L1* in the liver quickly causes non-alcoholic steatohepatitis (NASH) in mice [2, 9]. All of these results indicate that NFE2L1 is essential for maintaining the homeostasis of lipid and carbohydrate metabolism.

Interestingly, research has shown that NFE2L1 glycosylation mediates its cleavage and nuclear entry [10]. O-glycosylation in the Neh6L domain of NFE2L1 can reduce the transcriptional activity of NFE2L1 [11]. Additionally, in HepG2 cells, NFE2L1 deletion can cause cell death under glucose deprivation [12]. These studies suggest that NFE2L1 might be a key factor in regulating glucose metabolism homeostasis by sensing glucose levels. AMP-activated protein kinase (AMPK) plays an essential role in regulating energy metabolism [13, 14]. Our previous research showed that loss of NFE2L1 can disrupt AMPK signaling pathway, and NFE2L1 disrupts the activation of AMPK signaling induced by metformin (MET) [15]. However, MET promotes the degradation of NFE2L1 in an AMPK-independent manner [15], indicating the function of NFE2L1 as the upstream regulator of AMPK signaling.

Previous studies have suggested that NFE2L1 is essential for maintaining the homeostasis of glycose and lipid metabolism. And knockout of *NFE2L1* triggers NASH in liver, the most important metabolic organ, indicating that NFE2L1 is deeply involved in liver metabolism. Therefore, in this study, liver-derived HepG2 cell line was used to explore the effects of NFE2L1 deficiency on glucose metabolism. The results revealed the dual function of NFE2L1 as a glucose sensor and regulator of glucose metabolism. At the same time, we also identified the mechanism by which NFE2L1 regulates metabolism homeostasis.

## Materials and methods

### Cell lines, culture, and transfection

The cell lines used in this study are shown in Table S1. Cells were grown in DMEM supplemented with 10% (v/v) FBS. The experimental cells were transfected using Lipofectamine® 3000 Transfection Kit for 8 h and then allowed to recover from transfection in fresh medium. The kit information is presented in Table S1.

### Expression constructs and other plasmids

The expression constructs for NFE2L1 and LKB1 were constructed by cloning the target sequences from the full-length CDS sequences of *NFE2L1* and *LKB1* into the *pLVX-EGFP-puro* vector. The primers used for these expression constructs are listed in Table S1.

### Establishment of lentiviral-infected cell lines

Lentiviruses for the infection of HepG2^EGFP^ and HepG2^LKB1^ cells were packaged in HEK293T cells. HEK293T cells (5 × 10^5^) were seeded in six-well plates and were transfected with three plasmids (1 μg of *pMD2.G*, 2 μg of *psPAX2*, and 3 μg of either *pEGFP* or *pLKB1::EGFP* in 1 mL of transfection volume) when the cell confluence reached 80%. The medium was collected and filtered using a 0.45 μm sterile filter to obtain the virus after 36–48 h. The virus was used to transduce target cells, and puromycin (100 μM) was used to screen the infected cells.

### Cell viability assay

The experimental cells were seeded in 96-well plates and processed according to the experimental design. The cytotoxic effects of the indicated compounds on experimental cells were determined using the Cell Counting Kit-8 (CCK8) [16]. The absorbance at 450 nm was measured using a microplate reader (SpectraMax iD5, USA). The kit information is presented in Table S1.

### Cellular reactive oxygen species (ROS) staining

Experimental cells were allowed to grow to reach an appropriate confluence in six-well plates and then incubated in serum-free medium containing 10 μM 2′,7′-Dichlorodihydrofluorescein diacetate (DCFH-DA) [17] at 37 °C for 20 min. Thereafter, the cells were washed three times with serum-free media before the green fluorescent images were obtained by microscopy.

### Glucose uptake assay

The uptake of 2-Deoxy-2-[(7-nitro-2,1,3-benzoxadiazol-4-yl)amino]-D-glucose (2-NBDG), a fluorescent glucose analog, was used to visualize the uptake capacity of glucose by living cells. Experimental cells were incubated in serum-free medium containing 20 μM 2-NBDG at 37 °C for 10 min. Thereafter, the cells were washed three times with serum-free media before the green fluorescent images were obtained by microscopy.

### RNA isolation and real-time qPCR

Total RNA was isolated from experimental cells using the RNAsimple Kit. Then, 1 μg of total RNA was added to a reverse-transcriptase reaction to generate the first strand of cDNA using the RevertAid First Strand Synthesis Kit. The synthesized cDNA served as a template for qPCR with distinct primers, which were present in the GoTaq® qPCR Master Mix. The mRNA expression level of *β-actin* was selected as the optimal internal standard control. The primers used for qPCR and kit information are shown in Table S1.

### Western bloting (WB), Co-Immunoprecipitation (Co-IP), and protein deglycosylation reactions

Total protein extraction and WB were performed as previously described [15]. Co-IP was also performed as previously described [18]. Before visualization by WB, the deglycosylation reactions of samples with Endo H in vitro were carried out as described previously [19]. The information of all antibodies used in this study is shown in Table S1.

### Transcriptome sequencing

The cultured cells (HepG2^shNC^ and HepG2^shNFE2L1^) were lysed using Trizol, and transcriptome sequencing was performed by the Beijing Genomics Institute (BGI) with the order number of F21FTSCCKF2599_HOMdynrN. The samples were analyzed using the BGISEQ-500 platform. The transcriptome sequencing results are shown in Table S2.

### Metabolomics testing

The HepG2^shNC^ and HepG2^shNFE2L1^ cells were digested and counted, and cell pellets were obtained by low-speed centrifugation. Cell samples were immediately frozen in liquid nitrogen. Metabolomics testing was performed by the Metabolomics and Systems Biology Company, Germany; the order number was POCMTS2016006. The samples were measured with a Waters ACQUITY Reversed Phase Ultra Performance Liquid Chromatography device coupled with a Thermo-Fisher Exactive mass spectrometer. Three replicates were extracted and measured for each cell type.

### Electron microscopy

The adherent cells were digested with trypsin and fixed with 2.5% glutaraldehyde. The ultrastructure of the cells was examined using a transmission electron microscope (JEM-HT7700 model, Hitachi, Japan) at 5 μm, 1 μm, and 500 nm scales.

### Seahorse metabolic analyzer

The OCR of the cells was measured using the Seahorse XF Cell Mito Stress Test Kit. The experiment was performed according to the manufacturer’s instructions. The OCR was measured using a Seahorse metabolic analyzer to characterize the cell’s oxidative phosphorylation level. The information on Kit is shown in Table S1.

### Assays of ATP and lactate levels in cells

The ATP and lactate levels in cells were determined according to the instructions of the ATP assay kit and lactic acid assay kit. The information on Kits is shown in Table S1.

### Statistical analysis

The data are presented as a fold-changes (mean ± S.D.), each of which represents at least three independent experiments performed in triplicate. Significant differences were statistically determined by either Student’s t-tests or multiple analyses of variance. The statistical significance was defined by symbols; * indicates *p* < 0.05, whereas *n.s*. represents not significant.

## Results

### NFE2L1 deficiency disrupts cellular energy metabolism signals and induces cell death via glucose starvation

**Fig. 1:**
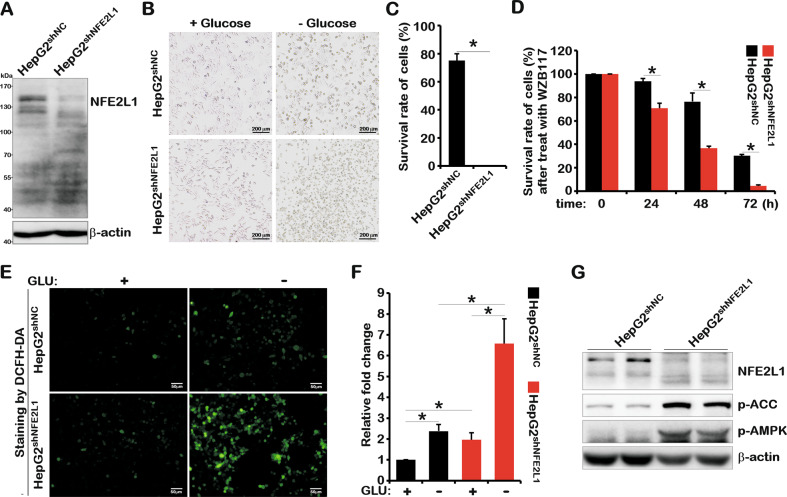
NFE2L1 knockdown causes HepG2 cells to be more sensitive to glucose starvation. **A** The total protein of HepG2^shNC^ and HepG2^shNFE2L1^ cells were collected, and then, the expression of NFE2L1 and β-actin were detected by western blotting (WB). The HepG2^shNC^ and HepG2^shNFE2L1^ cells were cultured in DMEM with or without glucose (2 g/L or 0 g/L) for 18 h, the morphology of cells was observed under a microscope (**B**), and the survival rate of cells was detected by a CCK8 kit (**C**). **D** The HepG2^shNC^ and HepG2^shNFE2L1^ cells were treated with WZB117 (200 μM), and the survival rate of cells was detected at 0, 24, 48, and 72 h using a CCK8 kit. The HepG2^shNC^ and HepG2^shNFE2L1^ cells were cultured in DMEM without glucose for 6 h, and then, the reactive oxygen species (ROS) in cells were staining with DCFH-DA (10 μM) for 20 min and imaged by microscopy (**E**); the intensity of fluorescence was quantitated (**F**). The scale is 50 μm. **G** The total protein of HepG2^shNC^ and HepG2^shNFE2L1^ cells were collected, and the expression of NFE2L1, pAMPK, pACC, and β-actin were detected by WB. *n* ≥ 3, *p < 0.05.

Previous studies have shown that mice with NFE2L1 overexpression or knockout exhibit diabetes-like phenotypes [6–8]. MET has been involved in regulating the function of NFE2L1 [15]. These studies indicated the involvement of NFE2L1 in maintaining glucose metabolism homeostasis. To further explore the relationship between NFE2L1 and glucose metabolism, a HepG2 cell line with *NFE2L1* knockdown was constructed (Fig. 1A). The glucose deprivation experiment results showed that HepG2 cultured with medium lacking glucose and HepG2 with *NFE2L1* deficiency were more susceptible to cell death (Fig. 1B, C). Here, WZB117 [20], a glucose uptake inhibitor, was used to test the effects of impaired glucose uptake on cell survival. The results showed that compared with control group (HepG2^shNC^), the *NFE2L1*-knockdown cells (HepG2^shNFE2L1^) exhibited poor survival rate when treated with WZB117 (Fig. 1D), which indicates that loss of NFE2L1 might disrupt the homeostasis of energy metabolism.

As a regulator of redox homeostasis, NFE2L1 could function to mediate oxidative stress. The results showed that the ROS level in the control group (HepG2^shNC^) was increased by approximately two-fold upon glucose deprivation for 6 h. In *NFE2L1*-knockdown cells (HepG2^shNFE2L1^), ROS levels were increased by approximately three-fold, and cell shrinkage was observed (Fig. 1E, F). And *NFE2L1*-knockdown resulted in the obvious elevation of ROS level both in the presence and absence of glucose, indicating the high levels of oxidative damage in *NFE2L1*-knockdown cells.

AMPK, the core signal of energy metabolism, is the guardian of metabolism and mitochondrial homeostasis [21]. Western blotting (WB) results showed that knockdown of *NFE2L1* significantly increased the phosphorylation level of AMPK and its downstream protein acetyl-CoA carboxylase alpha (ACC) [22] (Fig. 1G), indicating that the cells might have been in a starvation state due to the lack of NFE2L1 in HepG2^shNFE2L1^ cells. These results suggest that NFE2L1 functions by sensing the energy state of the cells.

### Glycolysis promotes glycosylation of NFE2L1 and NFE2L1 deficiency promotes glucose absorption

**Fig. 2:**
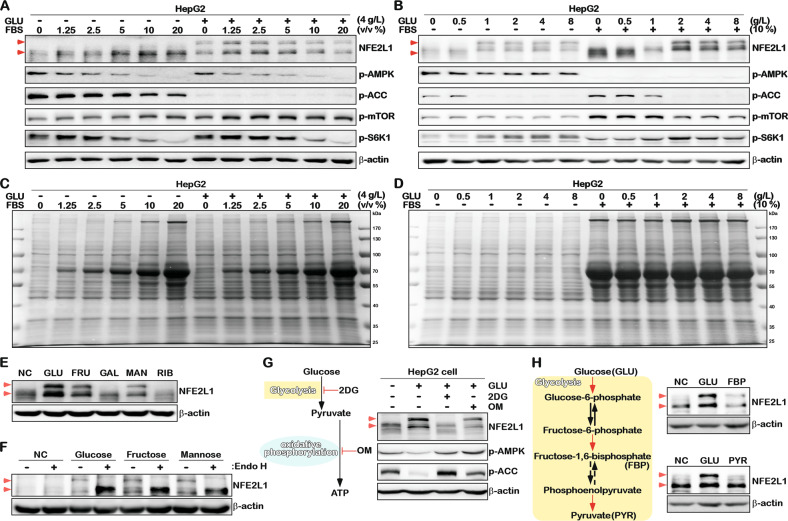
NFE2L1 is a glucose-sensitive protein. **A** HepG2 cells were cultured in DMEM medium with different volume ratios of FBS (0, 1.25, 2.5, 5, 10, 20%), with or without glucose (4 g/L or 0 g/L) for 16 h, and the total protein was collected, then, the expression of NFE2L1, p-AMPK, p-ACC, p-mTOR, p-S6K1, and β-actin were detected by WB. **B** HepG2 cells were cultured in DMEM medium with different concentrations of glucose (0, 0.5, 1, 2, 4, or 8 g/L), with or without FBS (10% or 0%) for 16 h, and the total protein was collected; then, the expression of NFE2L1, p-AMPK, p-ACC, p-mTOR, p-S6K1, and β-actin was detected by WB. **C** The total protein change in the sample in (**A**), based on staining with Coomassie brilliant blue. **D** The total protein change in the sample in (**B**) based on staining with Coomassie brilliant blue. **E** HepG2 cells were subjected to glucose starvation for 4 h and then cultured in DMEM with glucose (GLU; 2 g/L), fructose (FRU; 30 mM), galactose (GAL; 30 mM), mannose (MAN; 30 mM), and ribose (RIB; 30 mM) for 4 h. The total protein was collected and the expression of NFE2L1 and β-actin were detected by WB. **F** HepG2 cells were cultured in DMEM with GLU (0 g/L), GLU (2 g/L), FRU (30 mM), or MAN (30 mM) for 4 h, the total protein was collected, and Endo H was used to deglycosylate the glycated proteins in vitro; then, the expression of NFE2L1 and β-actin were detected by WB. **G** HepG2 cells were subjected to glucose starvation for 4 h and then treated with GLU (2 g/L), GLU (2 g/L) + 2-deoxy-D-glucose (2DG; 20 mM), or GLU (2 g/L) + oligomycin (OM; 10 μM), and the total protein was collected after 4 h; then, the expression of NFE2L1, p-AMPK, p-ACC, p-mTOR, p-S6K1, and β-actin were detected by WB. **H** HepG2 cells were subjected to glucose starvation for 4 h and then treated with GLU (2 g/L), fructose-1,6-bisphosphate (FBP; 30 mM), or pyruvate (PYR; 30 mM) for 4 h, the total protein was collected, and then, the expression of NFE2L1 and β-actin were detected by WB.

Studies have shown that NFE2L1 can be activated by the fetal bovine serum (FBS)/mechanistic target of rapamycin kinase (mTOR)/sterol regulatory element binding transcription factor 1 (SREBF1) pathway [23]. Accordingly, the mTOR and AMPK signaling pathways can be affected by NFE2L1 overexpression or knockdown [15], indicating the correlation between NFE2L1 and energy metabolism. Therefore, NFE2L1 alterations in response to the changes of glucose and FBS concentrations were tested, and the results showed that FBS could upregulate the total protein content of NFE2L1 and that glucose could affect the location of NFE2L1 protein on the SDS-PAGE (Fig. 2A, B). The level of p-AMPK gradually decreased with the increase of serum both in the presence and absence of glucose (Fig. 2A). The increased glucose slightly altered p-AMPK levels with and without FBS (Fig. 2B). And the p-mTOR appears to be more sensitive to FBS rather than Glucose (Fig. 2A, B).

Activation of AMPK and mTOR signals can be characterized by the phosphorylation level of ACC and phosphorylated-ribosomal protein S6 kinase B1 (p-S6K1) expression, respectively. In fact, as the results showed, p-AMPK was more sensitive to FBS in HepG2 cells (Fig. 2B), whereas p-ACC was more sensitive to glucose (Fig. 2A). Interestingly, the level of p-S6K1 increased at low concentrations of FBS and decreased when exposed to higher concentrations of FBS (Fig. 2A). With an increase in the glucose, the level of p-S6K1 was upregulated (Fig. 2B). In addition, FBS but not glucose promoted protein synthesis (Fig. 2C, D). It is important to note that the p-ACC level exhibited a strong negative relationship with the glycosylation of NFE2L1 (Fig. 2A, B). Together with the increase of p-ACC after NFE2L1 knockdown (Fig. 1G) and the decrease of p-ACC induced by NFE2L1 overexpression, all these results suggest that NFE2L1 might directly affect the enzymatic activity of AMPK.

Studies have shown that glycosylation regulates the activity of NFE2L1 [10, 11, 24, 25]. Here, the effects of five different monosaccharides on NFE2L1 protein were tested. The results showed that fructose (FRU) and mannose (MAN), similar to glucose (GLU), could induce the production of NFE2L1 with a larger molecular weight (Fig. 2E), which could be ascribed to the glycosylation of NFE2L1 protein, as revealed by the de-glycosylation experiment shown in Fig. 2F. In contrast, galactose (GAL) and ribose (RIB) had no effects on NFE2L1 (Fig. 2F).

The catabolism of carbohydrates mainly occurs with anaerobic glycolysis in the cytoplasm and aerobic oxidation in the mitochondria. In HepG2 cells, treatment with 2-Deoxy-D-glucose (2DG, a glycolysis inhibitor) [26] effectively inhibited glycosylation of NFE2L1. Inhibition of the oxidative phosphorylation process using oligomycin (OM) [27] decreased the total expression level of NFE2L1 (Fig. 2G). Further results showed that fructose-1,6-bisphosphate (FBP), the intermediate product of the glycolysis, induced the glycosylation of NFE2L1, whereas the end product pyruvate (PYR) had no such effects (Fig. 2H). These results suggest that the glycosylation modification of NFE2L1 is regulated by glycolysis rather than oxidative phosphorylation.

**Fig. 3:**
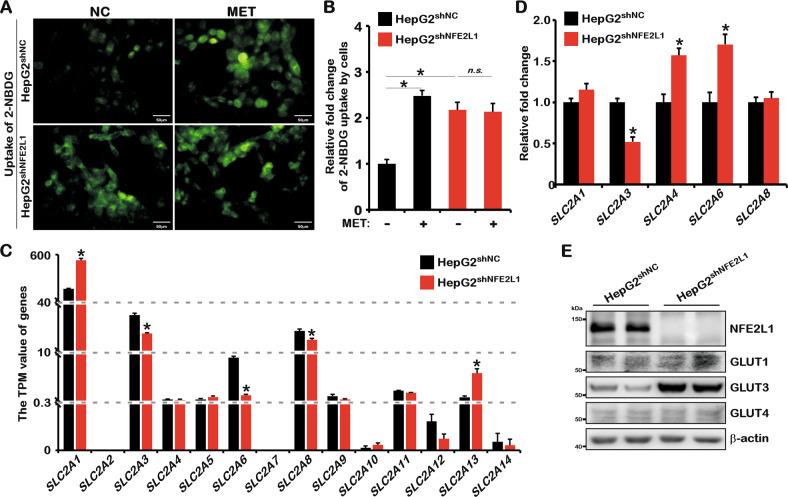
NFE2L1 affect the uptake of glucose by HepG2 cells. **A** HepG2^shNC^ and HepG2^shNFE2L1^ cells were treated with metformin (MET; 1 mM) for 12 h and then incubated in serum-free medium containing 20 μM of 2-NBDG at 37 °C for 10 min and the green fluorescent images were achieved by microscopy, the scale is 50 μm. **B** The statistics show the results in (**A**). **C** Transcripts Per Million (TPM) value of *SLC2A1*, *SLC2A2*, *SLC2A3*, *SLC2A4*, *SLC2A5*, *SLC2A6*, *SLC2A7*, *SLC2A8*, *SLC2A9*, *SLC2A10*, *SLC2A11*, *SLC2A12*, *SLC2A13*, and *SLC2A14* genes related to glucose uptake by cells; data originated from transcriptome sequencing. **D** Expression of *SLC2A1–9* genes in HepG2^shNC^ and HepG2^shNFE2L1^ cells as detected by qPCR. *SLC2A2*, *SLC2A5*, *SLC2A7*, and *SLC2A9* were not detected based on a lack of or extremely low expression, and *β-actin* was used as the internal control. **E** Expression of NFE2L1, GLUT1, GLUT3, GLUT4, and β-actin in HepG2^shNC^ and HepG2^shNFE2L1^ cells as detected by western blotting. *n* ≥ 3, *p < 0.05, ‘n.s.’ means ‘not significant’.

Our previous studies showed that NFE2L1 could be inhibited by MET, and the activation of AMPK signaling induced by MET could be disrupted by NFE2L1 knockdown [15]. Studies have shown that MET promotes glucose uptake in HepG2 [28]. Interestingly, here we found that NFE2L1 knockdown counteracted the effects of MET on glucose uptake in HepG2 (Fig. 3A, B). It should be commented here that glucose uptake was significantly increased by knocking down NFE2L1 that is likely the reason for a masked effect of MET. These data indicated that the promotion of glucose uptake mediated by MET in HepG2 might be related to NFE2L1. Subsequently, the changes in glucose transporter genes in the transcriptome data were assessed, and the data showed that after *NFE2L1* knockdown, *solute carrier family 2 member 1* (*SLC2A1/GLUT1*) and *SLC2A13* increased, whereas *SLC2A3*, *SLC2A6*, and *SLC2A8* decreased (Fig. 3C). The quantitative PCR (qPCR) results showed that *SLC2A3* expression was significantly reduced, whereas *SLC2A4* and *SLC2A6* were obviously increased (Fig. 3D). The *SLC2A2*, *SLC2A5*, *SLC2A7*, and *SLC2A9* could not be detected by qPCR, which might be due to the low or lack of expression of these genes. Next, the protein levels of glucose transporters were detected. The results showed that the protein expression level of GLUT3 was significantly increased after NFE2L1 knockdown (Fig. 3E), indicating that the increase in glucose uptake caused by NFE2L1 knockdown might be related to GLUT3. Studies have reported that low-glucose stress dramatically upregulated GLUT3 via the AMPK signaling pathway [29], which is consistent with the activated AMPK after NFE2L1 knockdown.

### NFE2L1 deficiency results in the reprogramming of glucose metabolism and exacerbates the Warburg effect

**Fig. 4:**
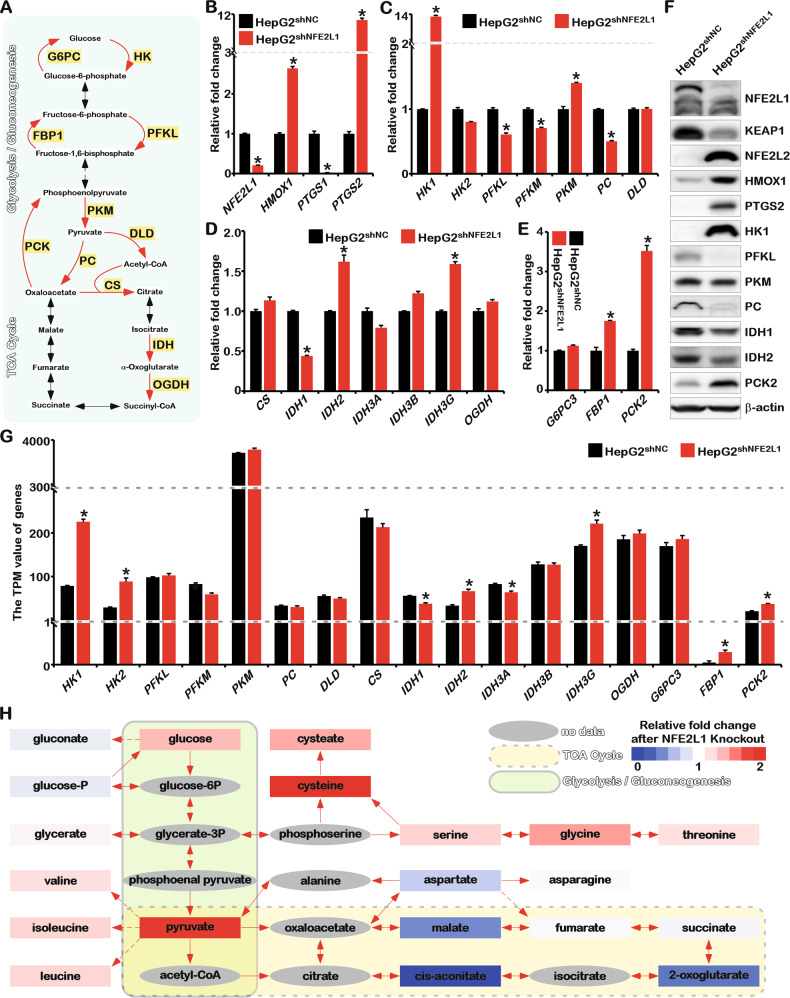
Knockdown of NFE2L1 leads to reprogramming of glucose metabolism. **A** The schematic diagram of glycolysis, gluconeogenesis, and TCA metabolism processes and key enzymes. **B** The expression of *NFE2L1*, *HMOX1*, *PTGS1*, and *PTGS2* genes in HepG2^shNC^ and HepG2^shNFE2L1^ cells as detected by qPCR; *β-actin* was used as the internal control. **C** The expression of *HK1*, *HK2*, *PFKL*, *PFKM*, *PKM*, *PC*, and *DLD* genes in HepG2^shNC^ and HepG2^shNFE2L1^ cells as detected by qPCR; *β-actin* was used as the internal control. **D** The expression of *CS*, *IDH1*, *IDH2*, *IDH3A*, *IDH3B*, *IDH3G*, and *OGDH* genes in HepG2^shNC^ and HepG2^shNFE2L1^ cells as detected by qPCR; *β-actin* was used as the internal control. **E** The expression of *NFE2L1*, *G6PC3*, *FBP1*, and *PCK2* genes in HepG2^shNC^ and HepG2^shNFE2L1^ cells as detected by qPCR; *β-actin* was used as the internal control. **F** The expression of NFE2L1, KEAP1, NFE2L2, HMOX1, PTGS2, HK1, PFKL, PKM, PC, IDH1, IDH2, PCK2, and β-actin in HepG2^shNC^ and HepG2^shNFE2L1^ cells as detected by western blotting. **G** The expression of key enzymes for glucose metabolism in the transcriptome data. **H** The results of the metabolome showed the effect of NFE2L1 on central metabolism. *n* ≥ 3, *p < 0.05.

The aforementioned results revealed the function of NFE2L1 in regulating glucose uptake. Next, the effects of NFE2L1 knockdown on the key rate-limiting enzymes involved in glycolysis, gluconeogenesis, and the tricarboxylic acid (TCA) cycle were tested (Fig. 4A). As shown in Fig. 4B, the increase of *heme oxygenase 1* (*HMOX1*) and *prostaglandin-endoperoxide synthase 2* (*PTGS2*) and the decrease of *PTGS1* were detected in the NFE2L1 knockdown cells [3]. *HK1*, which encodes the key enzyme in catalyzing the conversion of glucose to glucose-6-phosphate, was significantly increased. *PFKL* and phosphofructokinase (*PFKM*), which catalyze the conversion of fructose-6-phosphate to fructose-1,6-bisphosphate, were reduced. *PKM*, the enzyme critical for catalyzing phosphoenolpyruvate to pyruvate, was increased. No apparent change of *DLD*, the gene encoding the enzyme in catalyzing the entry of pyruvate into the TCA cycle, was detected. Expression of the *pyruvate carboxylase* (*PC*) was significantly reduced, indicating that the metabolic flow from glycolysis to the TCA cycle might be restricted (Fig. 4C).

The expression of genes encoding key enzymes involved in the TCA cycle was detected. The results showed that *citrate synthase* (*CS*) and *oxoglutarate dehydrogenase* (*OGDH*) exhibited no significant changes in mRNA levels. *Isocitrate dehydrogenase (NADP(*+*)) 1* (*IDH1*), which encodes the enzyme involved in catalyzing isocitrate to α-oxoglutarate, was reduced. Moreover, *IDH2* and *IDH3G* levels were upregulated (Fig. 4D). *Phosphoenolpyruvate carboxykinase 2* (*PCK2*) and *fructose-bisphosphatase 1* (*FBP1*), encoding the key rate-limiting enzymes in gluconeogenesis, were significantly increased, whereas no difference in *glucose-6-phosphatase catalytic subunit 3* (*G6PC3*) was detected (Fig. 4E), indicating that the gluconeogenesis process might be enhanced in the absence of NFE2L1.

The WB results showed that NFE2L1 knockdown could efficiently elevate the expression of NFE2L2, HMOX1, and PTGS2 and decrease Kelch-like ECH associated protein 1 (KEAP1) (Fig. 4F), which was consistent with our previous results [3]. The increased HK1 means enhanced glycolysis. The increased PCK2 indicated the enhancement of gluconeogenesis. Whereas the decreased PC implied the process from anaerobic glycolysis to aerobic oxidation was inhibited. Consistently, the transcriptome data also suggested that glycolysis and gluconeogenesis were enhanced after NFE2L1 knockdown in HepG2 cells (Fig. 4G). These results indicated that NFE2L1 deficiency might trigger the Warburg effect.

Analysis of metabolomic data showed that in the *NFE2L1*-knockout HepG2 cells, the content of glucose metabolism intermediate products, glucose, and pyruvate, was increased, and amino acids closely related to glycolysis intermediate products were significantly increased, including glycerate, valine, isoleucine, leucine, cysteate, cysteine, serine, glycine, and threonine (Fig. 4H). Malate, 2-oxoglutarate, and cis-aconitate, the intermediate products of the TCA cycle, were significantly reduced (Fig. 4H). These results implied that the absence of NFE2L1 produced an enhanced Warburg effect.

### NFE2L1 knockdown causes damage to mitochondrial function

**Fig. 5:**
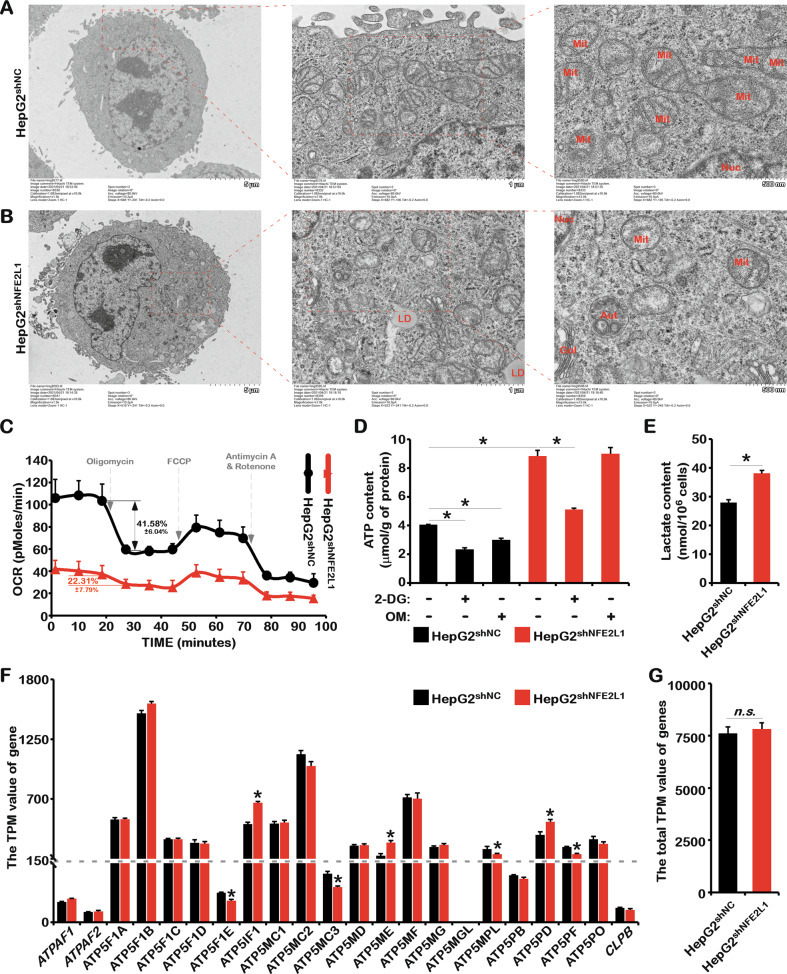
The effect of NFE2L1 knockdown in HepG2 cells on the morphology and function of mitochondria. The morphology of mitochondria in HepG2^shNC^ cells (**A**) and HepG2^shNFE2L1^ cells (**B**) were detected by electron microscopy. **C** The oxygen consumption rate (OCR) of HepG2^shNC^ and HepG2^shNFE2L1^ cells were measured with the Seahorse XF Cell Mito Stress Test Kit. **D** The HepG2^shNC^ and HepG2^shNFE2L1^ cells were treated with 2-deoxy-D-glucose (2DG; 20 mM) or oligomycin (OM; 10 μM) for 12 h, and the content of ATP in cells were detected with an ATP assay kit. **E** The content of lactate in HepG2^shNC^ and HepG2^shNFE2L1^ cells were detected with a lactic Acid assay kit. **F** The TPM values of *ATPAF1*, *ATPAF2*, *ATP5F1A*, *ATP5F1B*, *ATP5F1C*, *ATP5F1D*, *ATP5F1E*, *ATP5IF1*, *ATP5MC1*, *ATP5MC2*, *ATP5MC3*, *ATP5MD*, *ATP5ME*, *ATP5MF*, *ATP5MG*, *ATP5MGL*, *ATP5MPL*, *ATP5PB*, *ATP5PD*, *ATP5PF*, *ATP5PO*, and *CLPB* genes related to ATP synthesis in mitochondria in HepG2^shNC^ and HepG2^shNFE2L1^ cells; data originated from transcriptome sequencing. **G** The total TPM values of genes in (**F**). *n* ≥ 3, *p < 0.05, ‘n.s.’ means ‘not significant’.

Consistent with our results, previous studies have shown that the overexpression and knockout of NFE2L1 could alter glucose metabolism through different mechanisms, such as ROS, insulin secretion, and liver metabolism [6, 7, 12]. The TCA cycle in the mitochondria was inhibited once NFE2L1 was knocked down (Fig. 4), indicating the involvement of NFE2L1 on mitochondrial function. Electron microscopy results showed that NFE2L1 knockdown significantly reduced the number of mitochondria. The size of mitochondria with NFE2L1 knockdown seemed much smaller, and the ridge-like structure was significantly reduced (Fig. 5A, B). In NFE2L1-knockdown cells, accumulation of lipid droplets was observed, consistent with the lipid accumulation induced by specific NFE2L1 knockout in mouse liver [2]. Consistently, studies have shown that AMPK can promote mitochondrial division and mitochondrial autophagy [30, 31], suggesting that alterations to mitochondria caused by NFE2L1 knockdown might be related to the increased AMPK activity.

By testing the oxygen consumption rate (OCR) of the cells, it was found that oxygen consumption by the cells was reduced to approximately 40% of the control group after NFE2L1 knockdown (Fig. 5C). The oxygen consumption of control cells was reduced by 41.56 ± 6.04% after adding OM, an oxidative phosphorylation inhibitor. However, in the NFE2L1-knockdown cells, it was reduced by 22.31 ± 7.79% (Fig. 5C) indicating that the proportion of adenosine triphosphate (ATP) produced by mitochondrial respiration was significantly reduced after NFE2L1 knockdown. In the control group, the ATP content was reduced after treatment with 2DG or OM (Fig. 5D). In addition, the lactate content significantly increased after NFE2L1 knockdown (Fig. 5E). These results indicated that mitochondrial function was severely impaired after NFE2L1 knockdown.

In cells lacking NFE2L1, although mitochondrial function was impaired and the oxidative phosphorylation process was inhibited, the ATP content increased more than two-fold (Fig. 5D). This means that compared with those in the control group, cells lacking NFE2L1 needed to consume more glucose to produce ATP through glycolysis. This might be the reason why the lack of NFE2L1 led to increased cellular glucose uptake and cell death induced by glucose deprivation. No significant differences in genes related to ATP synthesis were observed (Fig. 5F, G) despite the effect of NFE2L1 deficiency on mitochondrial characteristics, indicating that NFE2L1 might indirectly regulate the total amount of ATP. It is worth noting that NFE2L1 knockdown could still activate AMPK in the presence of high ATP concentrations (Fig. 1G), suggesting that the influence of NFE2L1 on AMPK might occur through interfering the perception of ATP and/or adenosine monophosphate (AMP) by AMPK in cells.

### NFE2L1 interacts with AMPK and inhibits its phosphorylation via Serine/Threonine Kinase 11 (STK11/LKB1)

**Fig. 6:**
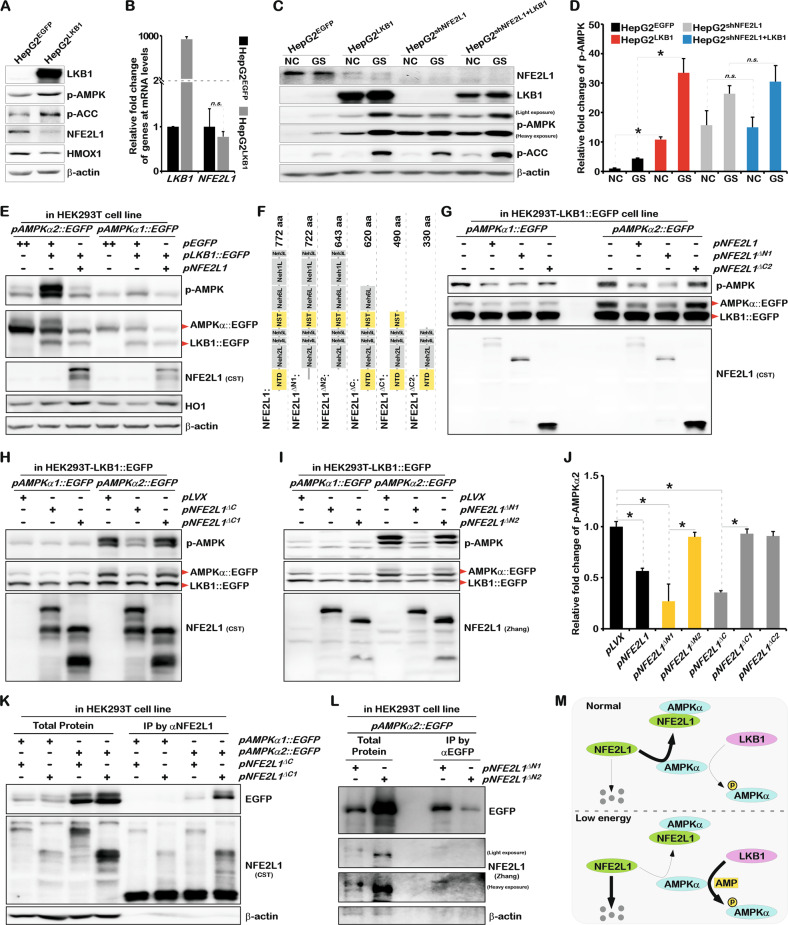
NFE2L1 disrupts the phosphorylation of AMPK mediated by LKB1 by directly interacting with AMPK. **A** The expression of LKB1, p-AMPK, p-ACC, NFE2L1, HO1, and β-actin in HepG2^EGFP^ and HepG2^LKB1^ cells were detected by WB. HepG2^EGFP^ and HepG2^LKB1^ cells were constructed via lentiviral infection. **B** The expression of *LKB1* and *NFE2L1* in HepG2^EGFP^ and HepG2^LKB1^ cells was detected by qPCR, with *β-actin* used as the internal control. **C** HepG2^EGFP^, HepG2^LKB1^, HepG2^shNC^, and HepG2^shNFE2L1+LKB1^ cells were treated with glucose starvation (GS) for 4 h, and the expression of NFE2L1, LKB1, p-AMPK, p-ACC, and β-actin were detected by WB. **D** Relative fold-changes in p-AMPK in (**C**). **E** The *pAMPKα1::EGFP* and *pAMPKα2::EGFP* were co-transfected with *pEGFP*, *pLKB1::EGFP*, *pNFE2L1* plasmids into HEK293T cells, respectively. And the expression of p-AMPK, EGFP, NFE2L1, LKB1, HO1, and β-actin were detected by WB. **F** Schematic diagram of NFE2L1 truncated protein. The *pAMPKα1::EGFP*, *pAMPKα2::EGFP* were co-transfected with *pNFE2L1*, *pNFE2L1*^*ΔN1*^, *pNFE2L1*^*ΔC2*^ (**G**) or *pLVX*, *pNFE2L1*^*ΔC*^, *pNFE2L1*^*ΔC1*^ (**H**) or *pLVX*, *pNFE2L1*^*ΔN1*^, *pNFE2L1*^*ΔN2*^ (**I**) into HEK293T-LKB1::EGFP cells, respectively. And the expression of p-AMPK, EGFP, and NFE2L1 were detected by WB. **J** Statistical analysis of the changes of p-AMPKα2 in (**G**–**I**). **K** The *pAMPKα1::EGFP* and *pAMPKα2::EGFP* were co-transfected with *pNFE2L1*^*ΔC*^ and *pNFE2L1*^*ΔC1*^ plasmids into HEK293T cells, respectively. And the total protein was collected with non-denaturing lysate buffer after 48 h. The specific antibody for NFE2L1was used for Co-IP experiments; then EGFP, NFE2L1 and β-actin were detected by WB. **L** The *pAMPKα2::EGFP*, *pNFE2L1*^*ΔN1*^, *pNFE2L1*^*ΔN2*^ plasmids were co-transfected into HEK293T cells, and the total protein was collected with non-denaturing lysate buffer after 48 h. The specific antibody for EGFP was used for Co-IP experiments; then EGFP, NFE2L1, and β-actin were detected by WB. **M** The model diagram of NFE2L1 involved in regulating AMPK signaling. *n* ≥ 3, *p < 0.05.

In the low-energy state, the ATP content in the cell decreases while the AMP content increases, which could induce LKB1 to phosphorylate AMPK [32–34]. The overexpression of LKB1 activated AMPK signaling and significantly inhibited the protein expression of NFE2L1 but showed no obvious effects on the mRNA level of *NFE2L1* (Fig. 6A, B), indicating the concurrence of NFE2L1 reduction and phosphorylated AMPK increase. To verify the effect of NFE2L1 on LKB1-induced AMPK phosphorylation, LKB1-overexpressing and NFE2L1-knockdown HepG2 cells were constructed. In cells with NFE2L1 deficiency, the overexpression of LKB1 did not further increase AMPK phosphorylation (Fig. 6C, D). Glucose starvation (GS) experiment results showed that GS incubation for 2 h significantly increased AMPK phosphorylation, and GS could still further increase AMPK phosphorylation after LKB1 overexpression (Fig. 6C, D). The cumulative effects of LKB1 overexpression and GS treatment on AMPK phosphorylation levels suggested that the response of AMPK to low-energy states might not completely depend on LKB1. In the NFE2L1-knockdown cells, with an increase in basal AMPK phosphorylation, although GS treatment further increased AMPK phosphorylation, the cumulative effect of LKB1 overexpression and GS treatment disappeared (Fig. 6C, D), suggesting that NFE2L1 indeed affected the phosphorylation modification of AMPK by LKB1. Consistently, the co-expression experiments showed that overexpression of NFE2L1 could significantly inhibit the phosphorylation of AMPK by LKB1, especially AMPKα2 (Fig. 6E).

Subsequently, truncated proteins of NFE2L1 were used to investigate the structure-dependent inhibition of AMPK by NFE2L1 (Fig. 6F). In HEK293T^LKB1::EGFP^ cell line, which stably overexpressed LKB1::EGFP protein, AMPKα1, and AMPKα2 were co-expressed with NFE2L1 and the truncated proteins. The results showed that NFE2L1^ΔN1^, the truncated NFE2L1 protein without the 1–50aa region and lost its membrane-bound structure, could still inhibit the phosphorylation of AMPK (Fig. 6G). NFE2L1^ΔC2^, which lacked 442aa on the C-terminal of NFE2L1, lost its inhibitory effect on AMPK (Fig. 6G). And the NFE2L1^ΔC^, lacking the Neh3L and Neh1L domains of NFE2L1, could inhibit AMPK, whereas the NFE2L1^ΔC1^, which lacking the Neh3L, Neh1L, and Neh6L domains of NFE2L1, failed to inhibit the phosphorylation of AMPK (Fig. 6H). These results suggest that Neh6L is an essential domain involved in mediating AMPK inhibition by NFE2L1.

Our previous study revealed that the inhibition of NFE2L1 protein by MET mainly relied on its NTD region. Interestingly, it was found that despite retaining the Neh6L region, the NFE2L1^ΔN2^, which missing the NTD region, also lost its inhibitory effect on AMPK (Fig. 6I, J). Thus, both the NTD and Neh6L of NFE2L1 were identified as necessary for its inhibition of AMPK. Subsequently, the Co-IP experiments showed that NFE2L1^ΔC1^ with the absence of the Neh6L region could still function to interact with AMPK (Fig. 6K), while NFE2L1^ΔN2^ that lacking the NTD region lost the ability to interact with AMPK (Fig. 6L). These results suggest that NFE2L1 binds to AMPK through the NTD region and mediates the inhibitory effect on AMPK via the Neh6L region.

## Discussion

NFE2L1 is essential for maintaining intracellular redox homeostasis, which is critical for cells to maintain normal metabolic processes. The increased glucose uptake caused by NFE2L1 knockdown might be related to the elevated GLUT3 expression (Fig. 3). However, the interaction between NFE2L1 and GLUT3, the transporter with the highest affinity for glucose [35], had not been explored. It is worth mentioning that the glycosylation and deglycosylation of NFE2L1 are necessary to activate its transcription factor activity to maintain redox homeostasis [19]. Meanwhile, the glycosylation of NFE2L1 strictly depended on glycolysis (Fig. 2E–H). These results suggest that NFE2L1 functions as a key factor in mediating glucose homeostasis and redox homeostasis in cells.

Studies have shown that NFE2L1 is sensitive to environmental nutrients [23]. However, the growth of cells lacking NFE2L1 depended almost solely on glucose. By detecting the key rate-limiting enzymes involved in glucose metabolism and the proteomics of glucose metabolites, we found that the loss of NFE2L1 significantly inhibited the oxidative phosphorylation process and enhanced glycolysis and gluconeogenesis (Fig. 4). Obviously, NFE2L1 deficiency in HepG2 triggered the enhancement of the Warburg effect, which might be the reason for the occurrence of spontaneous NASH that can finally develop into HCC in the liver of *NFE2L1*-knockout mice [2]. Here, we observed that the knockdown of NFE2L1 caused mitochondrial damage (Fig. 5). OM, an inhibitor of oxidative phosphorylation, barely inhibited ATP production in HepG2^shNFE2L1^ cells, indicating that these ATP were rarely produced by oxidative phosphorylation (Fig. 5D).

Interestingly, mitochondrial damage is considered to be the main source of excess ROS which could reversely cause mitochondrial damage [36]. The negative regulation of AMPK signaling by ROS has been analyzed [37]. However, the high ROS level and activated AMPK signals coexist in NFE2L1-knockdown cells (Fig. 1), suggesting that NFE2L1 may be directly involved in the negative regulation of AMPK. Under normal circumstances, high levels of ATP can inhibit AMPK to maintain energy homeostasis. The co-existence of high ATP content and highly activated AMPK signals in HepG2^shNFE2L1^ cells revealed the negative feedback regulation of AMPK signals have been destroyed or invalidated.

As an environment-sensitive protein, NFE2L1 alters in response to various stresses, even environmental temperature changes [4, 38]. As a transcription factor NFE2L1 could directly regulate the expression of thousands of genes [39, 40]. And NFE2L1, with a half-life of approximately 0.5 h, is always undergoing dynamic synthesis and degradation [15]. These characteristics allow NFE2L1 to mediate the interaction and communication between internal homeostasis and the external environment. Here, we determined that the phosphorylation of AMPK induced by MET [15] and LKB1 (Fig. 6C) is dependent on the decrease of NFE2L1. Therefore, we believe that the binding and inhibition of AMPK by NFE2L1 may be a normal state in cells, and the degradation of NFE2L1 in low-energy state or other stress is a necessary prerequisite for the activation of AMPK phosphorylation (Fig. 6M). Researchers have found that MET activates AMPK through PEN2/ATP6AP1/v-ATPase/Ragularor/AXIN/LKB1 pathway [41], and AMP drives AXIN to directly tether LKB1 to phosphorylate AMPK [34]. According to these factors, the scientific questions involved need further exploration including the identification of the molecule that relieves the direct binding and inhibition of AMPK by NFE2L1 during AMPK activation, whether NFE2L1 is degraded by the lysosomal pathway, and whether the oxidative stress regulates energy metabolism through the NFE2L1-AMPK axis.

In summary, we found that NFE2L1 could act as a sensor and regulator of glucose homeostasis, and impaired NFE2L1 caused the reprogramming of glucose metabolism, damaged the mitochondria, and aggravated the Warburg effect. As a main regulator of redox homeostasis, NFE2L1 was identified as a critical factor to mediate the negative regulation of AMPK signaling by directly interacting with AMPK. The novel NFE2L1/AMPK signaling pathway discovered in this study not only revealed the underlying mechanism of NFE2L1-related metabolic diseases but also highlight the crosstalk between redox homeostasis and metabolism homeostasis. These findings could deepen the understanding of the molecular mechanism through which NFE2L1 regulates metabolism and redox homeostasis, which is valuable for targeting NFE2L1 to prevent or treat systemic diseases.

## Supplementary information

Supplementary Table 1

Supplementary Table 2

Reproducibility checklist

## Data Availability

All data are available in this manuscript and supplementary files.
